# Gallium nitride multichannel devices with latch-induced sub-60-mV-per-decade subthreshold slopes for radiofrequency applications

**DOI:** 10.1038/s41928-025-01391-5

**Published:** 2025-05-22

**Authors:** Akhil S. Kumar, Stefano Dalcanale, Michael J. Uren, James W. Pomeroy, Matthew D. Smith, Justin A. Parke, Robert S. Howell, Martin Kuball

**Affiliations:** 1 Center for Device Thermography and Reliability, H. H. Wills Physics Laboratory, University of Bristol, Bristol, UK; 2 Northrop Grumman Mission Systems, Linthicum, MD USA

**Keywords:** Electrical and electronic engineering, Electronic devices

## Abstract

Aluminium gallium nitride/gallium nitride (AlGaN/GaN)-based superlattice castellated field-effect transistors are a potential basis for high-power radiofrequency amplifiers and switches in future radars. The reliability of such devices, however, is not well understood. Here we report transistor latching in multichannel GaN transistors. At the latching condition, drain current sharply transits from an off-state value to a high on-state value with a slope less than 60 mV per decade. Current–voltage measurements, simulations and correlated electroluminescent emission at the latching condition indicate that triggering of fin-width-dependent localized impact ionization is responsible for the latching. This localization is attributed to the presence of fin-width variation due to variability in the fabrication process. The latching condition is reversible and non-degrading, and we show that it can lead to improvement in the transconductance characteristics of transistors, implying improved linearity and power in radiofrequency power amplifiers.

## Abstract (web summary)

A distribution in fin widths, due to variability in the fabrication process, can be used to study a latching condition in gallium nitride transistors in which drain current sharply transits from an off-state value to a high on-state value with a slope of less than 60 mV per decade.

## Main

Gallium nitride (GaN)-based high electron mobility transistors (HEMT) have revolutionized wireless and military communication due to their high output power and high frequency operability^1^. This has been largely due to material parameters such as high saturation velocity, good room temperature mobility and high critical electric field^2–5^. Innovations in field plate design have further enhanced the performance of GaN HEMTs, leading to powers of up to 40 W mm^−1^ at 4 GHz and 60% power added efficiency^6^. However, future applications require further improvements in output power to maintain signal integrity over long distances, with ambitious projects having already set targets to achieve 81 W mm^−1^ (ref. ^7^).

The aluminium gallium nitride/gallium nitride (AlGaN/GaN)-based superlattice castellated field-effect transistor (SLCFET) could potentially be used to achieve such high output power targets^8–10^. SLCFETs with up to ten stacked two-dimensional electron gas (2DEG) channels offer a ten times increase in charge carriers compared with single-channel GaN HEMTs. This architecture provides a low knee voltage suitable for large output voltage swing combined with a high current density. SLCFETs have demonstrated output power of more than 10 W mm^−1^ at 94 GHz with 12 V on the drain and a power added efficiency of more than 40%, supported by current density of 4.8 A mm^−1^ with minimal dispersion and current collapse^11^.

A reliability challenge at such high power is managing heat dissipation without increasing the device footprint. Enhancing power is feasible by increasing the drain voltage (*V*_DS_). However, impact ionization could degrade the characteristics of SLCFETs^12^. Another possible consequence is transistor latching where the device remains in the on state despite being biased in off-state gate bias (*V*_GS_). This effect is well known in silicon-based devices^13–16^, but the high bandgap of GaN minimizes impact ionization^5^. We have previously confirmed the occurrence of impact ionization in the on-state operation of SLCFETs^12^.

In this Article, we report impact-ionization-induced latching in GaN transistors, visible in the subthreshold region. When the device turns on from the off state, the latch effect manifests as a subthreshold slope (SS) that is steeper than the Boltzmann limit of 60 mV per decade at 300 K. The underlying physics differs from other phenomena reported for sub-60-mV per decade slope, including negative capacitance^17–19^, hot-electron transfer^20,21^, displacement charge transfer^22^ and side wall conduction^23^. Electroluminescent (EL) micrography shows a localized latching mechanism, occurring at the widest fin among more than 1,000 fins. Scanning electron microscopy (SEM) imaging identifies a distribution in the fin width, the impact of which is corroborated by self-consistent simulations. The latching effect is reversible and non-degrading.

Although a steep SS occurs in the subthreshold region of operation, when averaged over a distribution of fin widths or when operated at radiofrequencies, this should provide a gradual increase in drain current in the on state, manifesting in a flatter transconductance for better linearity^21^. The resulting negative threshold voltage shift, generally associated with steep SS, facilitates the application of larger input swing for higher output power^24^. High-performance radiofrequency operation of SLCFETs has previously been achieved at high *V*_DS_, where latching was certainly occurring but was not reported^9^. We show that the broad transconductance response in the latched condition results in an improvement in linearity. This steep SS and enhanced linearity, along with excellent reliability, could be used to improve radiofrequency performance of multichannel GaN power amplifiers^25,26^.

## Device structure

**Fig. 1:**
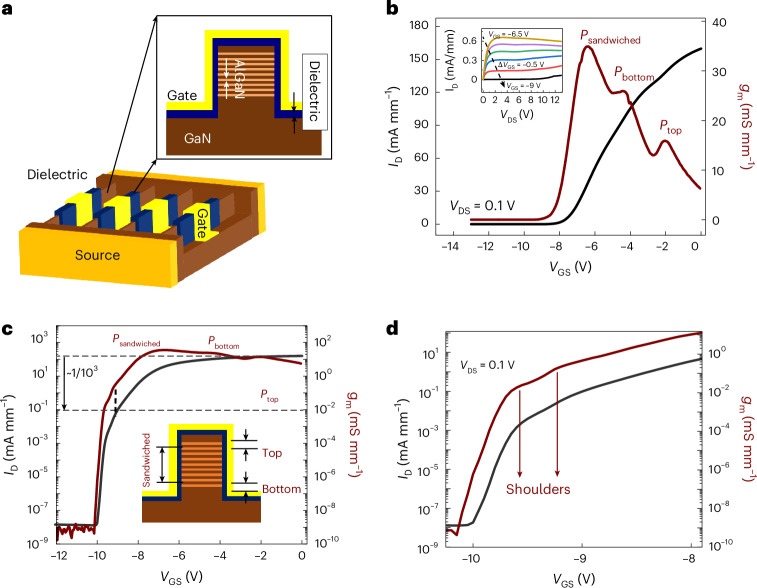
Schematics of a SLCFET along with its transfer characteristics. **a**, Schematics of a portion of a SLCFET having multiple (1,000s) of fins along with the cross-section of a single fin with its multiple conducting channels. **b**–**d**, Transfer characteristics and transconductance at *V*_DS_ = 0.1 V and Δ*V*_GS_ = 50 mV plotted in linear scale (**b**), here *P*_top_, *P*_bottom_ and *P*_sandwiched_ represent the *g*_m_ peaks originating from the topmost channel, bottommost channel and the channels in bewteen the two channels, respectively, and in log scale (**c**), and a zoomed-in portion of **c** between −10 V < *V*_GS_ < −8 V at *V*_DS_ = 0.1 V (**d**). Normalization was performed by dividing the measured current by device source width. Inset in **b**: *I*_D_–*V*_DS_ characteristic at high drain bias. Inset in **c**: the cross-section of a fin. The relative locations of top channel, bottom channel and the sandwiched channels are indicated by the arrows.

A schematic of a multifinger SLCFET is shown in Fig. 1a. An epitaxial grown material featuring ten periods of AlGaN/GaN superlattice structure created ten 2DEG channels. Side-gated electrostatic control on these channels was established using a fin structure, which was conformally coated with dielectric (SiN) and wrap-around gate metal. SiN/GaN forms type-II band alignment which ideally forms a hole-barrier-free interface for excellent device reliability^12,27^. A typical 60-µm-wide two-finger SLCFET possesses over 1,000 such fins between source and drain. The mean fin width was in the range of 30 to 100 nm, and the dielectric thickness was in the range 5 to 35 nm. The current–voltage (*I*–*V*) measurement in the linear regime is plotted in Fig. 1b. The multiple transconductance (*g*_m_) peaks in the on state (Fig. 1b) are attributed to the varying response of the different 2DEG channels in the fin to the gate electric field^12,28^, that is, top, sandwiched and bottom 2DEG channels. The inset of Fig. 1b shows the *I*_D_–*V*_DS_ characteristic of the SLCFET, which reveals a classic kink effect showing the presence of holes in the bulk of the semiconductor below the fin^29^. The subthreshold region of operation from Fig. 1c shows two distinct regions: (1) −10 V < *V*_GS_ < −9.5 V where *I*_D_ increases with a constant slope of around 80 mV per decade; and (2) −9.5 V < *V*_GS_ < −8 V where the *I*_D_ and *g*_m_ exhibits ‘stretching’ before entering the semi-on state. The presence of these two regions is uncharacteristic of conventional GaN HEMTs^26,30^. The logarithmic plot of *g*_m_ reveals shoulders in this stretched region (Fig. 1d). The location of the shoulders at more negative *V*_GS_ compared with that of the *g*_m_ peaks in the on state indicates channels with a more negative threshold voltage, |*V*_T_|, than is apparent in Fig. 1b. The value of *I*_D_ at these *g*_m_ shoulders was approximately three orders lower than *I*_D_ at *V*_GS_ = 0 V where all fins conduct.

## Fin-width variation

The subthreshold *g*_m_ shoulders may originate from variations in width between more than 1,000 fins. A distribution in fin width (with a deviation around a mean) leads to stretching of *I*_D_ and *g*_m_ in the subthreshold region due to a gradual sequential pinch-off of the different fins, owing to the width dependency of *V*_T_. These shoulders could originate from fins at the tail of this width distribution. The single sharp SS for *V*_GS_ < −9.5 V should then correspond to that of the outlier fin when other fins are depleted. Fabrication processes, involving lithography and dry etching, contribute to width variation. Adjacent fins can also merge to form wider fins.

**Fig. 2:**
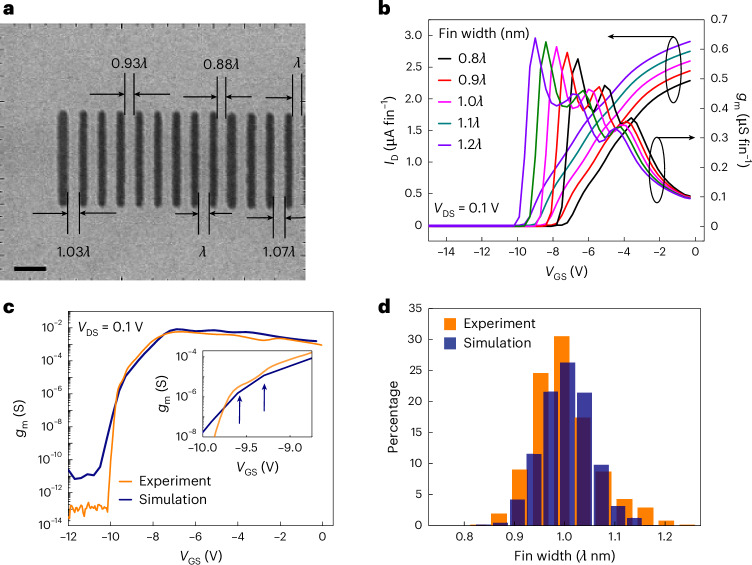
Technology computer-aided design modelling of fin-width variation to match experimental and simulated subthreshold characteristics. **a**, SEM imaging showing the fin-width variation. **b**, Simulated transfer characteristics for different fin widths. **c**, Matching of the experimental and simulated subthreshold *g*_m_. Inset: the experimental subthreshold *g*_m_ shoulders from Fig. 1(d) being compared with the simulated *g*_m_. The model is calibrated such that experimental *g*_m_ shoulders and the change in slope of simulated *g*_m_ have almost the same *V*_GS_. This matching is indicated by the two arrows. **d**, Comparison of the simulated Poisson distribution of 1,000 fin widths with the experimentally obtained width distribution. The plot shows number of fins (in percentage) as a function of absolute fin width. Here the value of one on the *x* axis corresponds to the mean fin width, *λ*.

To verify the hypothesis of fin-width variation, critical dimension scanning electron microscopy (CD-SEM) was performed. Figure 2a shows the SEM image of a part-processed SLCFET, before ohmic and gate metal contact deposition, revealing a non-uniformity in fin width. The effect of fin-width variation was modelled by solving self-consistently the transport and Poisson’s equation using Silvaco Atlas 3D. The AlGaN/GaN in each superlattice period was defined to be 8 nm/16 nm thick, respectively, as per ref. ^31^. For the efficacy of simulation and to avoid non-convergence, modelling was performed on a fin with six channels. The generality of results irrespective of number of channels is explained in ref. ^12^. The model was calibrated with gate-length of 0.25 µm assuming a mean fin width, *λ* nm, resulting in *V*_T_ of −8 V, as observed experimentally (Fig. 1b). Practically, this mean fin width, *λ*, was designed to be in the sub-100-nm range. Fin widths between 0.8*λ* and 1.2*λ* nm in steps of 2 nm were simulated, keeping other device parameters identical. Figure 2b shows the effect of different fin widths on the simulated *I*_D_ and *g*_m_ (per fin), where the three peaks along increasing positive *V*_GS_ correspond to the four sandwiched channels, and the bottom and top channels, respectively. As expected, larger fin widths lead to more negative *V*_T_. For overall effect, 1,000 fins were distributed, using different probability distributions, with *λ* nm as the mean fin width and different standard deviations, *σ*. A Poisson distribution with *σ* of 0.05*λ* nm, shown in Fig. 2d, resulted in a reasonable agreement with the experimental subthreshold *g*_m_ in the stretched-out region (−9.5 V < *V*_GS_ < −8 V), as illustrated in Fig. 2c. The inset in Fig. 2c captures the change in slope of simulated *g*_m_ (marked by arrows) at almost the same *V*_GS_ as the subthreshold *g*_m_ shoulders observed experimentally. This simulation verifies the change in slope to be from a few fins at the widest end of the distribution. Hence, the subthreshold *g*_m_ shoulders can be ascribed to the widest fins at the tail of the distribution. The simulated SS in the −10.5 V < *V*_GS_ < −10.0 V region was shallower than the experimental value. This can be explained by the actual distribution having integral numbers of fins with a cutoff corresponding to the widest fin, whereas the simulated distribution was continuous and had a long tail associated with numbers of fins less than one. In Fig. 2d, the simulated Poisson distribution is compared with the experimental distribution by equalizing the mean value to *λ* nm. Tails in the distribution towards the higher and lower widths are visible, as predicted by the simulation. The experimental distribution had a larger spread because it was compiled over multiple devices from multiple wafers, unlike the simulated data from a single device. This modelling exercise confirms that fin-width variation can indeed be responsible for *I*_D_ stretching and the *g*_m_ shoulders in the subthreshold region.

## Latching in SLCFETs

Latching occurs during high electric field operation when the channel switches from an off-state value to a high on-state value with an almost infinite slope. This characteristic has been reported for silicon-based devices such as silicon-on-insulator metal-oxide-semiconductor field-effect transistors, junction-less transistors and insulated-gate bipolar transistors^13–16,32^, for which, at high *V*_DS_, *I*_D_ is ‘latched’ to the on-current even when *V*_GS_ drops below *V*_T_. This is due to the floating body effect resulting from a self-limiting positive feedback loop from the storage of impact-ionization-generated holes. Below a particular *V*_GS_, the gate electric field overcomes this feedback loop and the device turns off with an almost vertical SS. No report of transistor latching in GaN devices is available, mainly due to the high bandgap in GaN (3.4 eV) compared with Si (1.1 eV), necessitating ‘hot’ electrons with much higher energy to trigger impact ionization. However, the trigate fin structure in SLCFETs with sub-100-nm fins would exhibit higher electric fields than conventional GaN HEMTs, which is evident from the high electron temperature estimated from spectroscopy of the Bremsstrahlung photons^12^. Although impact ionization has been demonstrated in multichannel GaN SLCFETs, there is no report of latching.

**Fig. 3:**
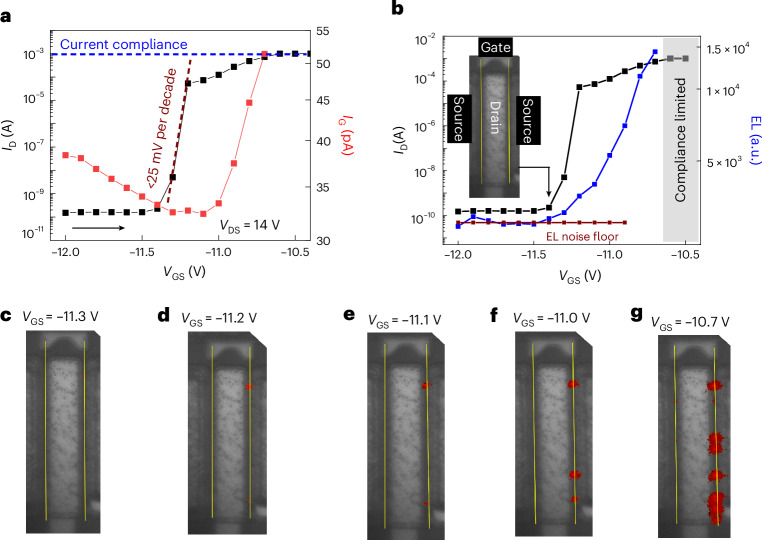
Current–voltage and simultaneous electroluminescense measurements at *V*_DS_ = 14 V, Δ*V*_GS_ = 100 mV and exposure time = 30 s. **a**, *I*_D_–*V*_G_ and *I*_G_–*V*_GS_ as *V*_GS_ is swept from −12 V to −10.5 V during the latching process. A 30-s step time ensured sufficient exposure for the microscope camera and resulted in sufficiently high signal-to-noise ratio to avoid weak localized emission being corrupted by the system noise. The current compliance of 1 mA prevented degradation of the device during the long step time. **b**, Summed EL as a function of *V*_GS_. Inset: EL micrograph at *V*_GS_ = −11.4 V. **c**–**g**, EL micrograph as a function of *V*_GS_ sweep for *V*_GS_ = −11.3 V (**c**), *V*_GS_ = −11.2 V (**d**), *V*_GS_ = −11.1 V (**e**), *V*_GS_ = −11.0 V (**f**) and *V*_GS_ = −10.7 V (**g**). The measurements were performed with a lens of numerical aperture = 0.6. The measurements of the EL spectrum in ref. ^9^. showed a minimum wavelength of approximately 500 nm. Consequently, the optical resolution of this measurement was around 500 nm (0.6 × wavelength/numerical aperture).

Figure 3 shows characterization of latching using *I*–*V* and EL measurements. In Fig. 3a, *I*_D_–*V*_GS_ and *I*_G_–*V*_GS_ are measured, with photoemission micrography performed at every bias point to check for hot-electron-induced EL emission. As *V*_GS_ is swept from off state into semi-on state, *I*_D_ rises sharply with SS of ≤25 mV per decade. Further sweeping shows the semi-on-state behaviour whereby *I*_D_ increases relatively slowly with *V*_GS_. In this same voltage range, the gate current *I*_G_ rises during the gradual increase of *I*_D_, presumably due to impact-ionization-generated holes migrating to the gate. The summed EL intensity from photoemission microscopy is plotted in Fig. 3b. In the off state, that is, *V*_GS_ = [−12 V, −11.4 V], the EL intensity is found to be limited by the noise floor. The noise floor of the system in the EL measurement was calculated by averaging the EL signal in the off state, as in Fig. 3b. The EL micrograph of the device biased at *V*_GS_ = −11.4 V (inset of Fig. 3b) shows no EL emission. As the device enters the subthreshold region at *V*_GS_ = −11.3 V, the summed EL intensity rises above the noise floor, coinciding with the sharp rise in *I*_D_. The EL images were processed to remove the noise and then overlaid as shown in Fig. 3c–g. At *V*_GS_ = −11.3 V (Fig. 3c), a faint EL spot originates slightly above the noise floor confirming the presence of localized hot carriers. At the next bias, that is, *V*_GS_ = −11.2 V, the summed EL intensity (Fig. 3b) increases further, and originates from the same spot (Fig. 3d). The peak intensity of the EL spot comes from the drain access region. However, the resolution is insufficient to determine whether the spot is near the gate or the drain edge. These observations: (1) the presence of a localized emission from hot carriers; and (2) the location of the EL spot in the drain access region, suggest that impact ionization might be occurring on a single (or few) fin(s) from among over a thousand fins. Its coincident appearance with the subthreshold step suggests that the fin with largest |*V*_T_| (that is, the widest fin in the distribution) has been ‘latched’. Further into the sweep, summed EL intensity increases monotonically with an increasing slope due to the combined effect of: (1) a gradual increase in intensity of each EL spot; and (2) the appearance of additional spots. At *V*_GS_ = −11 V (Fig. 3f), two more EL spots appear, attributed to the next two widest fin becoming latched on. The ratio of drain currents, that is, *I*_D_ at *V*_GS_ = − 11.0 V/*I*_D_ at *V*_GS_ = −11.2 V is 2.3 (<3), providing reasonable evidence for conduction happening in three fins of decreasing width as the sweep progresses. For *V*_GS_ > −11.2 V, the remaining fins, in reducing order of width, would experience the latch and subsequently enter conduction. This is evident in Fig. 3g, where there are multiple EL spots on both gates indicating a spatial randomness to the width distribution. Thus, EL measurements support the hypothesis that the steep increase in *I*_D_ is due to the high electric-field-induced latching on the widest fin.

## Reversibility, non-degradation and linearity

**Fig. 4:**
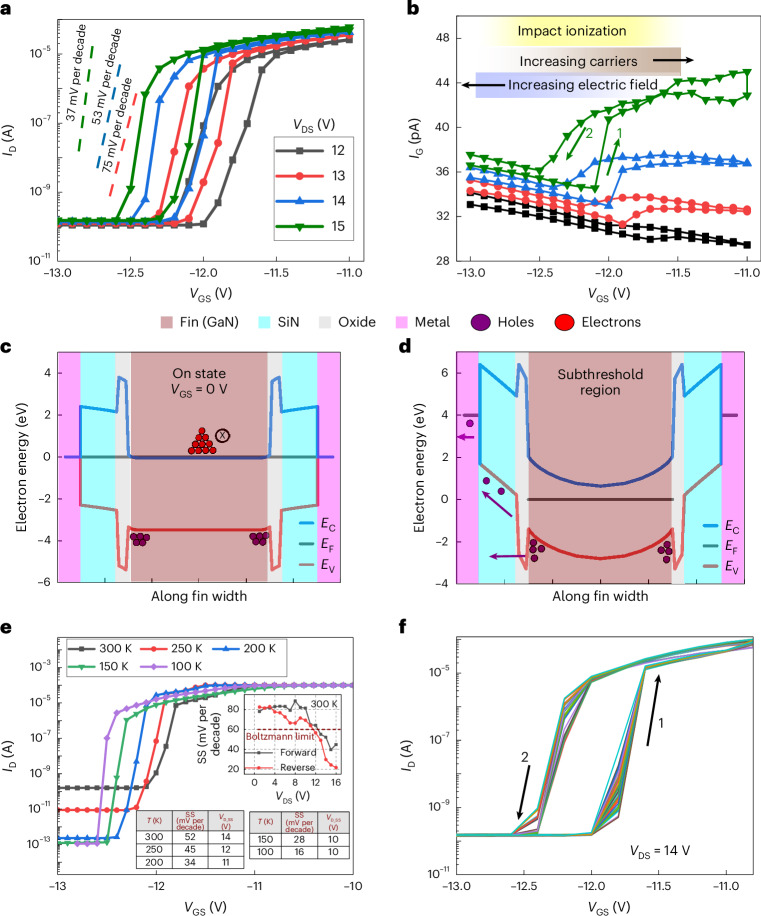
The origin and characterization of the latching effect. **a**, Transfer characteristics for varying *V*_DS_. **b**, *I*_G_–*V*_GS_ for the plots in **a**. **c**,**d**, Band diagram generated by self-consistent solution of drift-diffusion and Poisson’s equation in three dimensions cut along an AlGaN/GaN interface in the on state (**c**) and the subthreshold region (**d**). *E*_C_, *E*_F_ and *E*_V_ represent conduction band, electron fermi level and valence band energies, respectively. The holes and electrons generated during impact ionization are represented in the valence band and conduction band, respectively. **e**, *I*_D_–*V*_GS_ in subthreshold regime at the onset of SS < 2.3 kT q^−1^ for temperatures from 100 K to 300 K in steps of 50 K. To determine the onset, *V*_DS_ was swept in steps of 1 V from 6 V to 14 V at each temperature. The table indicates these *V*_DS_ onset values at which SS < 2.3 kT q^−1^ for each temperature. Inset: the variation of SS as a function of *V*_DS_ at 300 K. **f**, The latching process with hysteresis window over 30 runs with Δ*V*_GS_ of 200 mV.

This section explores the nature and mechanism responsible for the latching condition in SLCFETs, and then demonstrates that latching can be beneficial for radiofrequency power amplifiers (PA) linearity. In Fig. 4a, bidirectional sweeps in *V*_GS_ of a SLCFET for varying *V*_DS_ show that the SS becomes steeper as *V*_DS_ increases. Notably, although *I*_D_ increases abruptly with a slope <60 mV per decade (a slope of 2.3 kT q^−1^ or 60 mV per decade at room temperature, is expected for Boltzmann processes^33^), the process is reversible with a hysteresis window that shifts towards negative *V*_GS_ as *V*_DS_ increases. Two mechanisms are available in the literature to explain an abrupt increase in drain current under high field operation: (1) thermal runaway; and (2) avalanche multiplication (or impact ionization)^33^. The possibility of thermal runaway inducing the latching is explored in the Supplementary Information Section 1 and is not substantial. Electrothermal three-dimensional simulations using Ansys software at the latching condition showed a temperature rise of 283 °C, which is lower than that required to cause thermal breakdown in GaN transistors^34–37^. Impact ionization, if present at the latching condition, would manifest in a bell-shaped *I*_G_–*V*_GS_ plot^38,39^. As shown in Fig. 4b, *I*_G_ shows a jump at the point of latching, then peaks and subsequently reduces, which is consistent with a bell shape. This, along with its consistent *V*_DS_ dependency of *I*_G_, implies that the latching mechanism would most likely be induced by impact ionization. The sudden rise in *I*_D_ from the off state can be explained if some of the impact-ionization-generated holes on the widest fin get trapped/stored in its vicinity. This would shift *V*_T_, leading to positive feedback and an abrupt increase in both *I*_D_ and *I*_G_. To confirm the occurrence of impact ionization, *I*_D_–*V*_GS_ measurements at the latching condition were undertaken across a range of ambient temperatures. In Fig. 4e, the curve corresponding to the minimum *V*_DS_ (*V*_D_SS_) which results in SS < 2.3 kT q^−1^ is plotted. The table in the inset shows a positive temperature coefficient in *V*_D_SS_ at higher temperatures. This observation is consistent with the accelerating electrons attaining higher kinetic energy at lower temperatures, owing to larger mean-free-path, and increasing the rate of impact ionization. In the inset of Fig. 4e, similar monotonic reduction in SS is recorded as a function of *V*_DS_ at 300 K. This is again consistent with electrons gaining more kinetic energy at higher *V*_DS_, leading to enhanced rates of impact ionization.

Using the measured hole current in the gate and dimensions of the latched single fin, the minimum recovery time was approximately 1 μs (Supplementary Information Section 2). This constrains the possible location and trapping mechanism. Storage in deep levels in the GaN bandgap such as the dominant substitutional carbon acceptors at 0.9 eV above the valence band with time constants in the 1–100-s range explain the kink effect present in SLCFETs, but not the latching. Similarly, storage of free holes in the bulk of the fins is eliminated because a no-hole-barrier GaN/dielectric interface would transport holes through the dielectric in picoseconds (Supplementary Information Section 3). We propose the presence of a hole barrier at the dielectric/GaN interface storing holes for around a microsecond before they tunnel. Simulated band diagrams showing an additional oxide region between the fin and SiN, potentially from native oxide growth post fin etching and before the SiN deposition or from residual etch damage, are shown in Fig. 4c,d. The impact-ionization-generated electrons would drift into the drain terminal at high *V*_DS_. The holes generated would drift towards the source, resulting in a hole density along the entire length of the gate and hence a negative *V*_T_ shift. At sufficiently high *V*_GS_, an increased lateral electric field could cause the stored holes to tunnel into gate terminal (Fig. 4d). This emptying of holes, occurring on microsecond timescales, is faster than the sweep rate leading to the rapid rise/fall of *I*_D_ at the latching condition.

Impact ionization often results in permanent degradation and device failure. However, in some cases it is reversible without degradation. Reversible abrupt increases in current without permanent degradation have been reported in Si, MoS_2_ and GaAs devices^40–42^. However, similar data is unavailable for GaN transistors. Figure 4f explores the non-degradation at the latching condition with multiple bidirectional sweeps. The device exhibited consistent anticlockwise hysteresis without signs of degradation, validating the underlying mechanism of hysteresis in Fig. 4a to be storage/emptying of holes residing in the semiconductor.

**Fig. 5:**
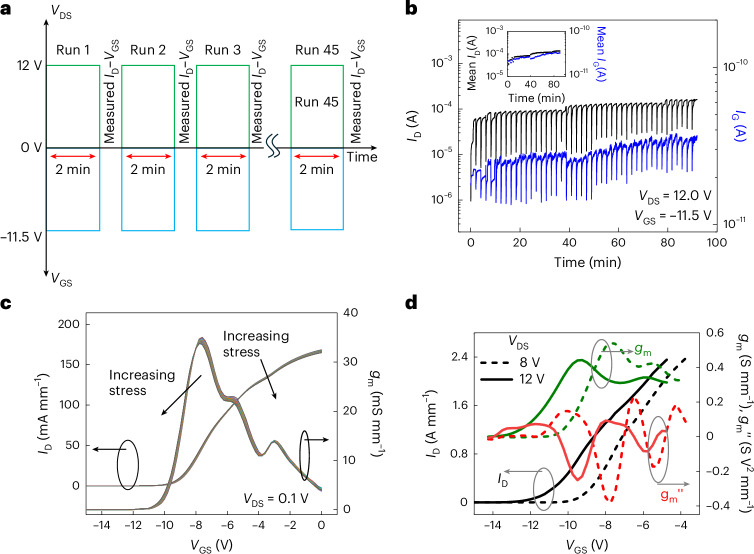
Reliability studies of SLCFET at the latching condition along with its practical application. **a**, Methodology adopted for stress testing of latching condition. The device was stressed for 90 min in steps of 2 min and *I*_D_–*V*_GS_ at *V*_DS_ = 0.1 V to monitor the device condition. **b**, The evolution of *I*_D_ and *I*_G_ as a function of stressing time at *V*_DS_ = 12 V and *V*_GS_ = −11.5 V. Inset: the mean value of *I*_D_ and *I*_G_ over each 2-min cycle. **c**, The monitored *I*_D_ and *g*_m_ as function of *V*_GS_ at *V*_DS_ = 0.1 V after each stress cycle in the latching condition. **d**, The *I*_D_, *g*_m_ and ${g}_{{\rm{m}}}^{{\prime\prime} }$ of the SLCFETs are plotted when biased at *V*_DS_ of 8 V (unlatched condition) and 12 V (latched condition). Current compliance for *I*_D_ was set to 200 mA. The normalization in **d** was performed by dividing the measured *I*_D_ by the active source width of the device, which was approximately 50% of entire width. This was to have a one-to-one comparison with similar fin-based devices in the literature, for example, ref. ^43^.

The analysis of device stability, reliability and the application of the latching condition in power amplifiers is shown in Fig. 5. The methodology followed is outlined in Fig. 5a with each cycle comprising a stressing and a condition-monitoring step. Figure 5b presents the *I*_D_ and *I*_G_ during the stressing period, with the mean value of each cycle in the inset. The plots reveal an insignificant change in *I*_D_ and *I*_G_, indicating no *V*_T_ instability associated with charge trapping and time-dependent dielectric breakdown. In Figure 5c, post stress *I*_D_–*V*_GS_ to monitor the on-state characteristics of the device indicate no *V*_T_ shift. The *I*_D_ and *g*_m_ characteristics show no sign of instability or reliability issues due to the latching condition.

The non-degradation observed is explained by the maximum rate of impact ionization coincident with the steep SS, which reduces as *V*_GS_ becomes less negative due to the lowering of effective electric field in the fins. This non-degrading nature of steep SS is also evident in Chang et al., where large-signal operations were performed on SLCFETs under d.c. conditions exhibiting latching^9^. Although other GaN-based transistors have demonstrated lower than 60 mV per decade, their performance and subthreshold reliability does not match that of SLCFETs^17–23^_._ This work suggests that latching is an inherent property of GaN finFETs where the etching of fin sidewalls forms a small hole barrier between the semiconductor and the SiN dielectric. This barrier would act as a hole reservoir on the microsecond timescale. Such a mechanism would not be applicable to a planar GaN HEMT and could explain why latching is not observed in those devices.

The effect of the latching condition on the radiofrequency linearity performance of SLCFETs has been analysed using d.c. *I*_D_–*V*_GS_ and transconductance, as shown in Fig. 5d. The same SLCFET was operated at *V*_DS_ of 8 V and 12 V corresponding to latched and unlatched conditions, respectively. A *V*_T_ shift of approximately −3 V for *V*_DS_ = 12 V operation was observed. This is attributed to all the 1,000+ fins entering the reversible and non-degrading latching condition, causing the *V*_T_ shift as explained as part of Fig. 4. In the latched condition, the *g*_m_ shows a reduction of the peak value by approximately 20%. However, this value is still comparable with state-of-the-art GaN radiofrequency devices^43^. Interestingly, the ${g}_{{\rm{m}}}^{{\prime\prime} }$_,_ which is a key parameter determining linearity, reduces by a factor of two, indicating a strong improvement in linearity. This ${g}_{{\rm{m}}}^{{\prime\prime} }$ has attained a reduction of approximately two times that of other multichannel GaN-FETs and six times that of GaN HEMTs^43^. This linearity improvement can be attributed to the latching effect whereby, as *V*_GS_ is swept from subthreshold to *V*_T_, all the 1,000+ fins get latched to a high on state. This shifts *V*_T_ and broadens the transconductance characteristics so that, as *V*_GS_ sweeps towards 0 V, the *I*_D_ increase will be more gradual than when all the fins are not in a high on state, that is, an unlatched state. Under radiofrequency conditions, where the cycle time is much faster than the hole residence time, the steep SS regime would not occur and there would be smooth negative shift in *V*_T_. The improved linearity and larger usable *V*_GS_ range will allow the application of larger input power, *P*_in_, giving higher output power, *P*_out_, as was seen experimentally in large-signal radiofrequency measurements by Chang et al.^9^. The effect on the power–linearity trade-off, caused by reduction in *g*_m_, is analysed in Supplementary Information Section 4.

## Conclusions

We have reported transistor latching in GaN-based transistors for which, at high drain bias operation, the drain current increases from off state to near on state with a subthreshold slope less than the Boltzmann limit of 60 mV per decade at 300 K. The latching process observable in subthreshold is a consequence of a distribution in fin widths, arising from fabrication-related process variation between the more than 1,000 sub-100-nm-width fins. This results in a width distribution with a peak at the mean value and with gradual tails towards the narrowest and widest fin, confirmed from SEM imaging. Using EL, *I*–*V* and self-consistent solutions of the transport and Poisson’s equation, the latching process was shown to be localized on individual fins starting from the widest fin. The latching was attributed to impact ionization on the widest fin, which shifts the threshold voltage to becoming more negative by the storage of holes generated by impact ionization within the fin. The latching was also shown to be reversible and non-degrading, and to result in improved transconductance characteristics consistent with improved radiofrequency linearity. This latch-induced steep subthreshold slope could be used to further improve the performance of SLCFET-based radiofrequency transistors.

## Methods

**Extended Data Fig. 1:**
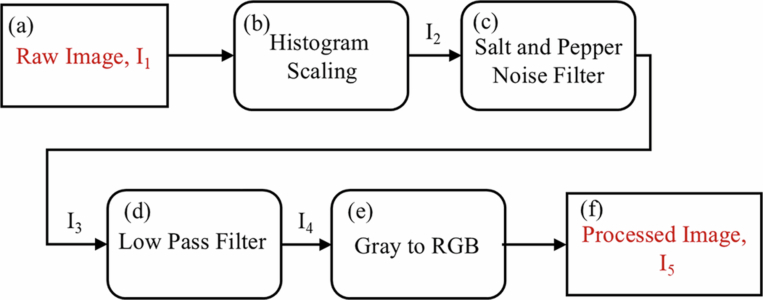
The step-by-step algorithm used to extract the EL signal from noisy signal. a, The greyscale raw image is obtained from EL micrography. b, Histogram scaling is performed in order to increase the EL intensity without pixel saturation. c, The noise signal which also scaled in (b) is reduced partially using Salt and Pepper noise filter. d, Low pass filtering is performed to help eliminate high frequency noise present in (c). e, The greyscale image is colour coded based on the intensity level of each pixel. f, The final processed EL image after filtering algorithm.

**Extended Data Fig. 2:**
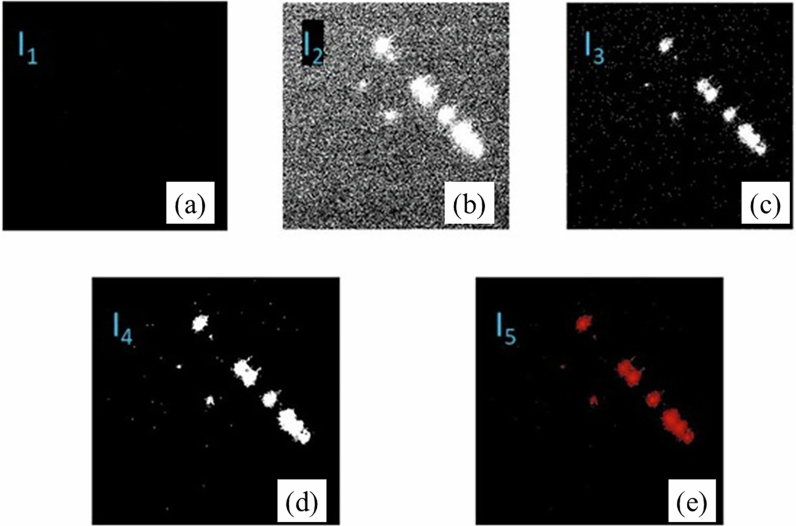
The result of imaging processing on one of the noisy images as per Extended Data Figure 1. a, The raw EL image in greyscale. b, Intensity-enhanced image using histogram scaling. c, Image after Salt and Pepper filtering. d, Image after low pass filtering. e, greyscale to RGB coded image.

The intensity of the EL signal shown in Fig. 3c–e was near the minimum detectable signal-to-noise ratio of the camera used. This is because each EL spot was emerging from individual fins with a current drive of only ~10 µA per fin during the emission process. Such low current drive resulted from the low concentration of hot electrons in the entire device during the latching process. To subtract the noise from the captured EL signal, image processing was performed as shown in Extended Data Figs. 1 and 2. The image was processed in MATLAB.

## Supplementary information

Supplementary Information Supplementary Sections 1–4 and Figs. 1–2.

## Data Availability

The detailed device structure is proprietary information of Northrop Grumman and cannot be made available. This specifically includes information on device geometry such as fin width, fin separation, fin height, dielectric thickness and so on. The data that was used to plot the figures within this paper are available from the corresponding author upon reasonable request.

## Code availability

The custom-developed codes for simulating SLCFETs using Silvaco’s Atlas solver may be available from the corresponding author, subject to clearance from Northrop Grumman. The critical device dimension will be presented as variables having values within a range to respect the proprietary rights of Northrop Grumman. The algorithm used in the MATLAB code for image analysis of the EL signal is shown in the Methods. The specific parameters used in the image-processing code can be made available from the corresponding author on request.
