# Supplementary material for: Gallium nitride multichannel devices with latch-induced sub-60-mV-per-decade subthreshold slopes for radiofrequency applications
Source: Nat Electron. 2025 May 22;8(6):510–7. doi: 10.1038/s41928-025-01391-5 (PMC12202481; doi:10.1038/s41928-025-01391-5)
Supplement: Supplementary file 1 — Supplementary Sections 1–4 and Figs. 1–2. [file 41928_2025_1391_MOESM1_ESM.pdf]

nature electronics

Supplementary information https://doi.org/10.1038/s41928-025-01391-5

Gallium nitride multichannel devices with latch-induced sub-60-mV-per-decade subthreshold slopes for radiofrequency applications

In the format provided by the authors and unedited

Approved for Public Release; NG24-2376; ©2024 Northrop Grumman Systems Corporation

Supplementary Document

1. Thermal runaway

3D electrothermal simulation is performed to determine the self-heating in a single fin of a SLCFET as shown in Fig. Supplementary Figure 1 (1). Initially, ATLAS simulation is performed on a single fin at the bias of V_{DS}=15 V and V_{GS}=- 5 V on a fin width of λ nm. Based on this model, the electric field is concentrated within 200 nm of the drain edge of the gate in this semi-ON state (Fig. Supplementary Figure 1 (2)). Volumetric Joule heating is applied in the ANSYS model to a 200 nm long block in the channel at this location. This is based on the model used by Middleton et al (https://doi.org/10.1109/LED.2019.2929424). The power dissipation in the single fin, obtained from experiemental I_D=50 μA at V_{DS}=15 V, is 0.7 mW which translated to peak temperature of 283 °C (Fig. Supplementary Figure 1 (3)). This temperaure is much lower than those reported in the literature for GaN HEMTs in safe operating regime[30- 32,42]. Hence, thermal runaway causing sharp subthreshold slope can be ignored.

Approved for Public Release; NG24-2376; ©2024 Northrop Grumman Systems Corporation

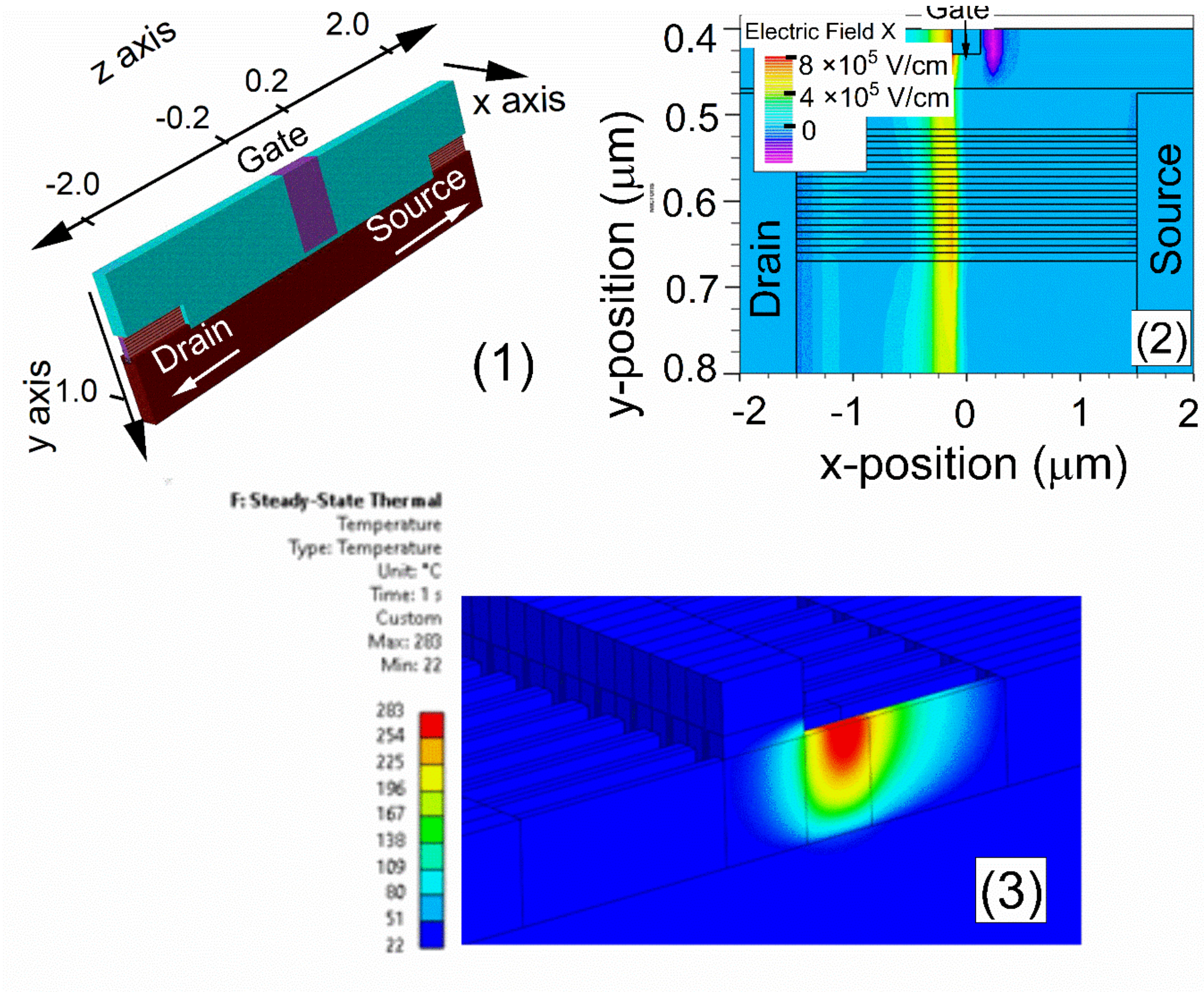

Supplementary Figure 1. Simulation to determine peak channel temperature in SLCFET at latching condition. 1) 3D schemtics of the device used for ATLAS simulation, 2) Electric field along an x-y plane at the drain end of gate, and 3) Temperature countour of self-heating of a single fin obtained from ANSYS.

2. Hole residence time, t_{empty}

An order of magnitude estimate for the minimum stored hole residence time under latch conditions can be made. We estimate that a hole density of ~10^{11} cm^{-2} within the device fin would causes the V_T shift of 0.25 V seen in Fig. 4(a) (as is the case for a typical planar GaN HEMT). For the latching condition to sustain with this charge density, the hole current, $I_{G\text{-}h}$ in gate leakage would need to be

$$I_{G-h} = \frac{\text{charge}}{t_{\text{empty}}} = \frac{B\ 10^{11}\ \times 1.6\times 10^{-19}\text{C/cm}^2}{t_{\text{emply}}} \text{ ------------------------------------ (A)}$$

where B is the active area where latch has occurred. The experimental component of hole current in gate leakage from Fig. 4(b) can be assumed to be the component resulting in the rise in I_G during latching. This is a current of ~5 pA. Experimentally, latch occurs in a single fin so the hole current density would flow through the sides of the

Approved for Public Release; NG24-2376; ©2024 Northrop Grumman Systems Corporation

fin, and in order for it to result in a V_T shift, the holes must extend along the entire gate length in the fin. Hence the latch area B would correspond to a gate length of 0.25 µm and fin width of ~100 nm. Using these values gives

$$t_{empty} \sim 1\ \mu s$$

This is the timescale in which the stored holes would empty from the gate at the point of steep subthreshold slope either by transport to the gate or by electron-hole recombination. Hence it represents the minimum hole residence time required to sustain latch.

3. Hole transit time, $t_{transit}$

Assuming no hole barrier on the sides of fin, the approximate transit time needed for holes from the semiconductor to transppport into the gate can be calculated from $t_{transit} \sim \frac{fin-width/2}{v_{holes}} = \frac{fin-width/2}{\mu_{holes} \times E_{lateral}}$, where $E_{lateral}$ is the lateral electric field under the gate and μ_{holes} is the mobility of holes in GaN. $E_{lateral}$ is approximated from the band diagram in Fig. 4(d) as the ratio of potential difference in half the fin to half the fin width. $t_{transit}$ is approximately 1 picosecond. Such a transit time results in gate leakage current as per

$$I_{G-hole} = \frac{hole\ density \times q}{t_{transit}} = \frac{10^{11}\ cm^{-2} \times 1.6\times10^{-19}\ C}{1\times10^{-12}\ s} = 10^4\ \text{A}/cm^2$$

Using the dimensions of gate as in B above, I_{G-hole} is approximately 1 µA, which is much higher than the measured value of I_G in Fig. 4(a). This confirms the presence of a hole barrier at the sides of fin.

4. Power-Linearity Tradeoff

As observed, in Fig. 5(d), the g_m peak shows a reduction by approximately 20 % together with a 2x reduction in g_m''. A figure-of-merit to evaluate the power-linearity tradeoff is the third-order intecept (TOI). TOI can be extracted from g_m and g_m'' using a Volterra series expansion as in Ref D1 and Ref D2 below. In the presence of non-linearity, the input-output relationship can be expressed as:

$$V_o = k_o + k_1 V_i + k_2 V_i^2 + k_3 V_i^3 + \cdots \text{ --------------------------------------- (D.1)}$$

where V_i , V_o and k_i are the input signal, output signal and coefficients of the Volterra series. For a sinusoidal input signal, $V_i = Acos(\omega t)$, the output power in dBm is

$$P_o = 10\ log_{10}((\frac{k_1 A + \frac{3}{4} k_3 A^3}{\sqrt{2}})^2 \frac{10^3}{R}) \text{ --------------------------------------- (D.2)}$$

where R is the load resistance. At TOI, $k_1 A = \frac{3}{4} k_3 A^3$ i.e $A_{TOI}^2 = \frac{4k_1}{3k_3}$. The output power at TOI becomes

$$P_{TOI} = 10\ log_{10}(\frac{2}{3}\frac{k_1^3}{k_3}\frac{10^3}{R}) \text{ --------------------------------------- (D.3)}$$

k_1^3/k_3 can be approximateted from eq. (D.1) as g_m^3/g_m''. Then P_{TOI} becomes

Approved for Public Release; NG24-2376; ©2024 Northrop Grumman Systems Corporation

$$P_{TOI} = 10\, log_{10}(4\frac{g_m^3}{g_m''}\frac{10^3}{R}) \text{ -- (D.4)}$$

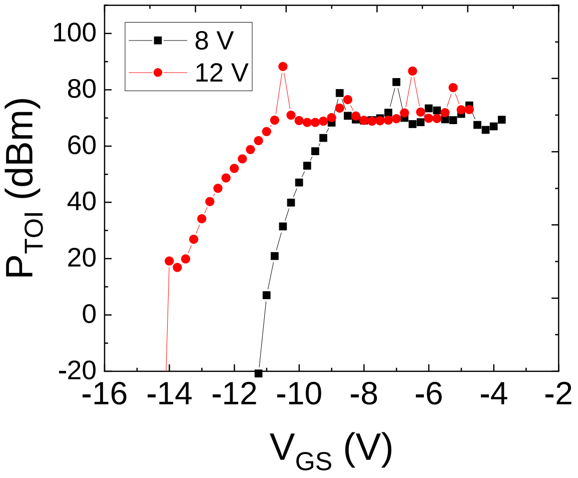

Supplementary Figure 2. Power-Linearity trade-off in SLCFET. P_{TOI} plotted for latched (V_{DS} = 12 V) and unlatched condition (V_{DS}=8 V).

Assuming load resistance of 50 Ω, the P_{TOI} has been plotted for the latched and unlatched condition of Fig. 5(d) as in Supplementary Figure 2. For -9 V < V_{GS} , the P_{TOI} is found to the preseved. In the range, -13 V < V_{GS} < -9 V, the P_{TOI} in the latched condition is much superior than in unlatched condition. Thus, the decrease in g_m-peak is compensated by the decrease on g_m” and does not affect the RF performance, but very usefully the usable input voltage range is increased by 2V.

References:

D1. Bailey, Michael Jon. "Intermodulation distortion in pseudomorphic HEMTs and an extension of the classical theory." *IEEE Transactions on Microwave Theory and Techniques* 48.1 (2000): 104-110.

D2. Li, Tao, Ravindra P. Joshi, and Romeo D. del Rosario. "Requirements for low intermodulation distortion in GaN-Al/sub x/Ga/sub 1-x/N high electron mobility transistors: a model assessment." *IEEE Transactions on electron devices* 49.9 (2002): 1511-1518.
